# Effect of the gut microbiota on frozen shoulder and potential mediating role of inflammatory cytokines: A Mendelian randomization study and mediation analysis

**DOI:** 10.1097/MD.0000000000044595

**Published:** 2025-09-26

**Authors:** Zhiyun Feng, Xiule Fang, Tao Ding

**Affiliations:** a The Affiliated Wuxi People’s Hospital of Nanjing Medical University, Wuxi, China.

**Keywords:** frozen shoulder, gut microbiota, mediation analysis, Mendelian randomization

## Abstract

Frozen shoulder (FS), characterized by chronic inflammation and joint fibrosis, remains etiologically unclear. Emerging evidence suggests that gut microbiota may modulate systemic inflammation via the “gut-joint axis,” yet causal links to FS are unverified. This study aimed to investigate GM-FS causality using Mendelian randomization and explore inflammatory cytokines as potential mediators. This study was conducted using genome-wide association study data from MiBioGen and IEU. Six cytokines were included in the mediation analysis. Inverse variance weighted served as the primary method, supplemented by weighted median and MR-Egger, with sensitivity analyses to ensure robustness. Among 119 bacterial genera, Butyrivibrio (OR = 0.922), Lachnospiraceae UCG004 (OR = 0.897), Lactobacillus (OR = 0.843), Parabacteroides (OR = 0.790), and Ruminococcaceae UCG003 (OR = 0.836) are protective against FS, while *Eubacterium coprostanoligenes* (OR = 1.172) is a risk factor (all *P* < .05). Mediation analysis revealed interferon -γ partially mediated the protective effect of Lactobacillus on FS (β = 0.170, *P* = .011). These findings advance the “gut-joint axis” theory and suggest novel therapeutic targets for FS, deserving further experimental validation.

## 1. Introduction

Frozen shoulder (FS), also known as adhesive capsulitis, is a chronic inflammatory disease characterized by shoulder pain, limited range of motion, and capsular fibrosis.^[1]^ Based on the characteristics of the disease process, FS can be divided into 2 categories: primary FS of unknown etiology, which is defined as spontaneous onset without known cause or trauma and accounts for more than 70% of clinical cases; and secondary FS, which is closely associated with trauma, surgery, or other pathology. Epidemiologic studies have shown that the prevalence in the general population worldwide is approximately 2% to 5%, with a significantly higher risk in women than in men between the ages of 40 and 60 years,^[2]^ while the prevalence in diabetic patients may be as high as 20%.^[3]^ Typical pathologic features include synovial hyperplasia, inflammatory cell infiltration, and impaired collagen deposition in the joint capsule, leading to a reduction in joint space volume of more than 50%.^[4]^ Although risk factors, such as sedentary lifestyle and autoimmune diseases, have been widely studied, the exact etiology remains unclear. Conventional treatments are based on nonsteroidal anti-inflammatory drugs, physical therapy, and intra-articular glucocorticoid injections; however, approximately 20% to 30% of patients do not respond well to conventional treatments and ultimately require arthrocentesis.^[5]^ In recent years, biologics targeting the inflammatory microenvironment and antifibrotic agents, such as pirfenidone, have entered clinical trials,^[6]^ but efficacy is still limited by a lack of understanding of the etiology. Emerging evidence suggests that FS may be associated with a systemic inflammatory imbalance: dysbiosis in the gut flora of patients with metabolic diseases such as diabetes and obesity may promote systemic inflammation through the “gut-joint axis,”^[7]^ while gut flora-derived short-chain fatty acids (SCFAs) may inhibit joint fibrosis by modulating Treg cell function.^[8]^ However, the causal relationship between the bacterial group and FS has not been proven by high-level evidence.

In recent years, the physiological regulation of gut microbiota (GM) as a “second genome” has been gradually revealed, and its “bone-muscle axis,” formed by metabolites, immune regulation and neuroendocrine pathways, profoundly affects bone remodeling and muscle homeostasis.^[9]^ Studies have shown that gut flora dysbiosis can lead to decreased bone density, sarcopenia and joint degeneration: on the one hand, flora metabolites such as SCFAs inhibit osteoblast differentiation and promote osteoclast mineralization through the activation of G protein-coupled receptors (GPR43/41)^[10]^; on the other hand, flora-derived secondary bile acids modulate muscle protein synthesis and bone mineralization through the Farnesoid X Receptor/Takeda G protein-coupled receptor 5 (FXR/TGR5) signaling axis.^[11]^ Clinical cohort studies have further confirmed that patients with osteoporosis and rheumatoid arthritis have a significantly higher abundance of Prevotella and a lower abundance of butyrate-producing bacteria in their intestinal flora.^[12]^ Notably, gut flora can also influence local inflammation by modulating the Th17/Treg cell balance: in a rat model of d-galactose induction, oral administration of Bifidobacterium fragilis significantly attenuated the inflammatory response by increasing Treg cells.^[13]^ Based on the “bone-gut axis” theory, intervention strategies targeting the flora have shown clinical potential. For example, probiotic (e.g. Lactobacillus) supplementation increases bone mineral density and attenuates inflammatory damage in collagen-induced arthritis in mice,^[14]^ and a high-fiber diet enriches SCFA-producing flora, increases circulating levels of butyrate, and directly represses genes related to muscle wasting, such as atrogin-1, MuRF-1.^[15]^ However, most of the existing studies have focused on systemic skeletal diseases or large joint degeneration, while there is an almost complete lack of attention to FS. This limitation may be due to 2 factors: firstly, the relationship between local pathological features of FS and systemic inflammation mediated by the gut flora has not yet been elucidated; secondly, how the metabolites of the gut flora can penetrate the “gut-synovial” barrier and act on the microenvironment of the shoulder joint remains to be mechanistically elucidated. However, recent animal studies have shown that high-fat diet-induced disturbances in the GM can induce shoulder joint degeneration in mice through the NF-κB pathway, and supplementation with SCFA-producing bacteria can inhibit interleukin (IL)-1β and tumor necrosis factor-α (TNF-α) expression in the synovial membrane of the shoulder joint through the butyrate-GPR43 axis and attenuate fibrosis in the FS.^[16]^ This suggests that the use of probiotics may be a novel therapeutic strategy for the treatment of shoulder disorders such as FS and rotator cuff injuries.

Mendelian randomization (MR) is a method of causal inference based on genetic variation.^[17]^ Its core idea is to use genetically determined instrumental variables (IVs) that are randomly assigned in nature to simulate the logic of randomized controlled trials and thereby elucidate the causal relationship between exposures. Single nucleotide polymorphisms (SNPs) that are strongly associated with exposure will be selected as genetic IVs. The relative effect of the association between the outcome and the exposure is calculated to infer causality. Because genetic variation is randomly distributed during meiosis, MR results are unlikely to be influenced by environmental factors that may confound the estimate.^[18]^ MR is particularly valuable in studies of GM and disease. Microbiota composition is highly regulated by host genetics (heritability of approximately 5–20%), and traditional observational studies are prone to time-dependent confounding factors such as diet and antibiotic use. However, MR analysis can mitigate these confounding factors to some extent.^[19]^ In this study, we conducted bidirectional MR analysis based on extensive genome-wide association study (GWAS) data on shoulder periarthritis and GM to explore the causal relationship between GM and shoulder periarthritis. Then, we used mediation analysis to investigate whether multiple inflammatory factors might mediate the causal relationship between GM and shoulder periarthritis.

## 2. Materials and methods

### 2.1. Study design

**Figure 1.**
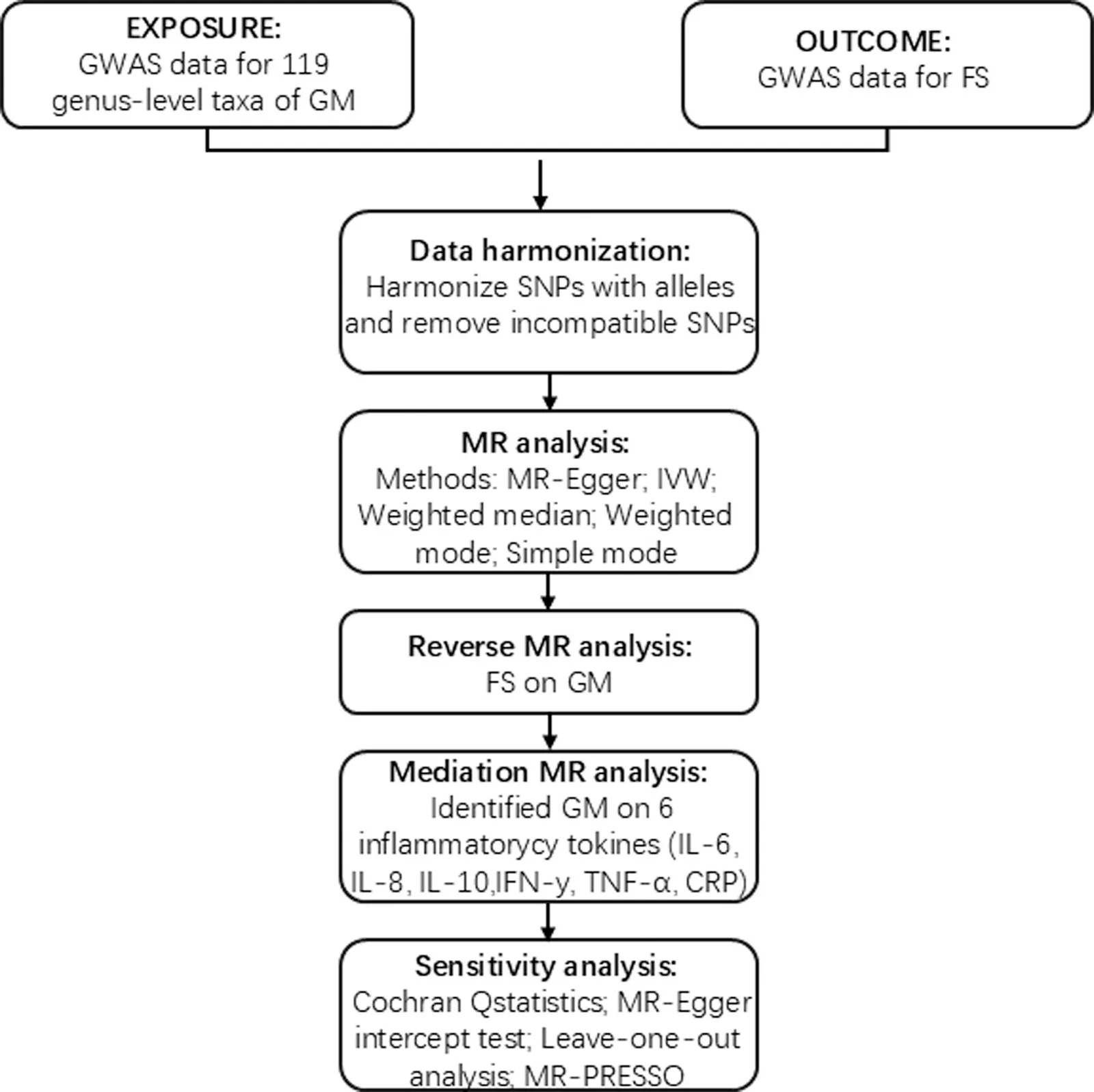
The flowchart of MR analyses between FS and GM. FS = frozen shoulder, GM = gut microbiota, GWAS = genome-wide association study, IFN-γ = interferon-γ, IL = interleukin, IVW = inverse variance weighted, MR = Mendelian randomization, MR-PRESSO = Mendelian randomization-pleiotropy residual sum and outlier, SNPs = single nucleotide polymorphisms, TNF-α = tumor necrosis factor-α.

**Figure 2.**
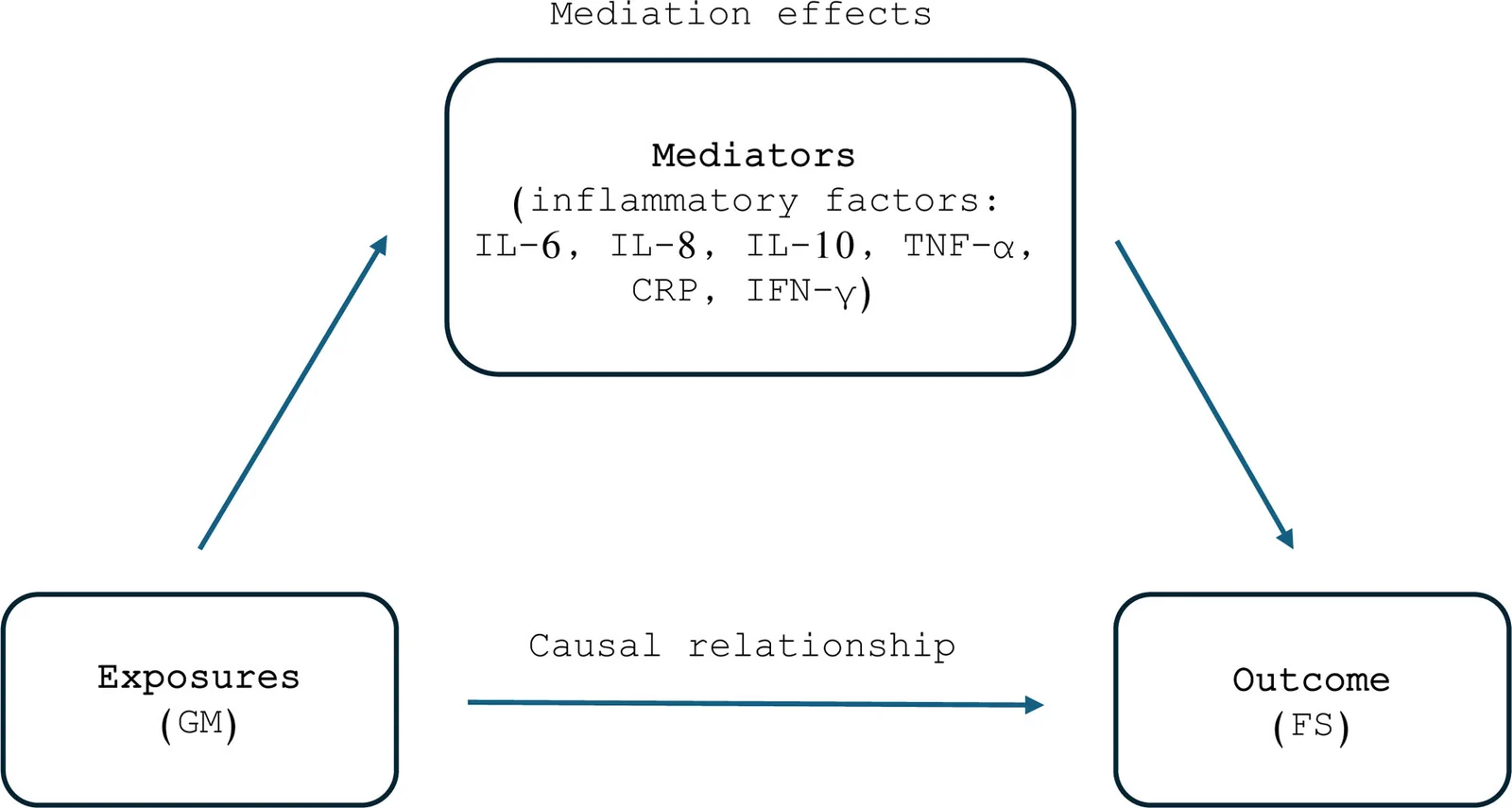
Diagrams illustrating associations for mediation MR analysis. FS = frozen shoulder, GM = gut microbiota, MR = Mendelian randomization.

The study design strictly followed the STROBE-MR guidelines^[20,21]^ for strengthening the reporting of observational studies using MR. Figures 1 and 2 show a flow chart of our study and the general process of mediation analysis. The MR analysis of the causal relationship between shoulder periarthritis and GM is based on the following assumptions: the selected IVs should be closely associated with exposure; IVs are independent of confounders associated with exposure and outcomes; and IVs influence outcomes only through exposure and not through direct associations. Overall, we used MR to identify which GM influence FS, and then selected 6 inflammatory factors that have been extensively reported to be associated with the pathogenesis of FS, including IL-6, IL-8, IL-10, interferon-γ (IFN-γ), C-reactive protein (CRP), and TNF-α,^[22–24]^ and conducted a mediation MR analysis to determine which inflammatory factors mediate the causal relationship between GM and FS. Finally, sensitivity analyses, including MR-Egger’s intercept test^[25]^ and Cochran’s *Q* test,^[26]^ were performed to validate the accuracy and robustness of the results. All data used in this study were obtained from publicly available databases and did not require additional ethical approval.

### 2.2. Data sources

In this study, GM genetic IVs were obtained from public databases, covering a total of 211 taxonomic units, including 9 phyla, 16 classes, 20 orders, 35 families, and 131 genera.^[27]^ This is the largest GWAS of GM composition to date aimed at identifying genetic variation in GM composition. The data are available online (https://mibiogen.gcc.rug.nl/). Among the 211 taxonomic units, 131 genera had an average abundance >1%, including 12 unidentified genera. Therefore, only 119 taxonomic units at the genus level were analyzed in this study. GWAS summary data for FS were extracted directly from the IEU GWAS database (https://gwas.mrcieu.ac.uk/), GWAS ID = ebi-a-GCST90000512, including 451,099 European participants and 15,184,371 SNPs.^[28]^ GWAS summary data for inflammatory cytokines were also obtained from the IEU GWAS database, with basic information shown in Supplementary File 1, Supplemental Digital Content, https://links.lww.com/MD/Q158.

### 2.3. Instrumental variable selection

SNPs significantly associated with 119 target bacterial genera were extracted based on GWAS data from the MiBioGen consortium (n = 18,340). A genome-wide significance threshold (*P* < 5 × 10^−^⁸) was set to control the false positive rate. However, due to the limited number of IVs obtained at this threshold, a broader and more commonly used threshold (*P* < 1 × 10^−^⁵) was used to obtain a relatively larger number of IVs, thereby providing more robust results. In addition, linkage disequilibrium pruning was performed to ensure the independence of each IV (*r*^2^ < 0.001, window size 10,000 kb).^[29]^ To further exclude weak IVs, the *F*-statistic (*F* = (β/SE)^2^) was calculated for each SNP, and weak IVs with *F* < 10 were excluded.^[30]^ Potential phenotypic associations of candidate SNPs were identified using the PhenoScanner database (http://www.phenoscanner.medschl.cam.ac.uk/), and multi-effect SNPs directly associated with known risk factors for shoulder periarthritis or confounders were excluded,^[31]^ and performed directional consistency correction for the effect alleles of exposure and outcome GWAS data. The final retained SNPs must meet the criteria of significant genetic association (*P* < 1 × 10^−^⁵, *F* ≥ 10), independence from confounders, and absence of pleiotropic pathways (MR-Egger intercept test *P* > .05).

### 2.4. Statistical analysis

In this study, inverse variance weighted (IVW) was used as the primary analysis method.^[32]^ To validate the robustness of the results, sensitivity analyses were performed using weighted median, MR-Egger, weighted mode, and simple mode.^[33]^ In the MR-Egger regression, the intercept obtained was used to assess horizontal pleiotropy (an intercept *P* > .05 was considered to indicate no significant bias). If the intercept was significantly different from 0, this indicated the presence of average pleiotropic effects of IVs on the outcome variable.^[25]^ Cochran’s *Q* test was used to assess heterogeneity among SNPs, and Mendelian randomization-pleiotropy residual sum and outlier (MR-PRESSO) was used to detect and remove outlier SNPs.^[34,35]^ Correction for multiple comparisons was performed using the Bonferroni method,^[36]^ with a significance threshold of *P* < .05/119 = 4.2 × 10^−4^. If the *P*-value was <4.2 × 10^−4^, it was considered statistically significant, and if the *P*-value was between 4.2 × 10^−4^ and .05, it was considered indicative of a possible causal relationship. To rule out the confounding effect of a single SNP on the results, a leave-one-out analysis^[37]^ was also performed. All analyses were performed using the TwoSampleMR and MR-PRESSO packages in R (v4.3.1).

### 2.5. Reverse-direction MR analysis

To rule out the reverse causal effect of FS on GM, this study also performed reverse-direction MR analysis. Reverse direction MR analysis reverses the 2 variables, that is, FS is considered as exposure and GM as outcome. This is because if a reverse causal relationship exists, the conclusions of the original analysis may be unreliable.

### 2.6. Ethics statement

All data for GM and FS were collected from public database GWAS, and ethical reviews were approved by their respective study institutions. All analyses were conducted online, and no new ethical approval was required.

## 3. Results

### 3.1. Causal effects of GM on FS

**Figure 3.**
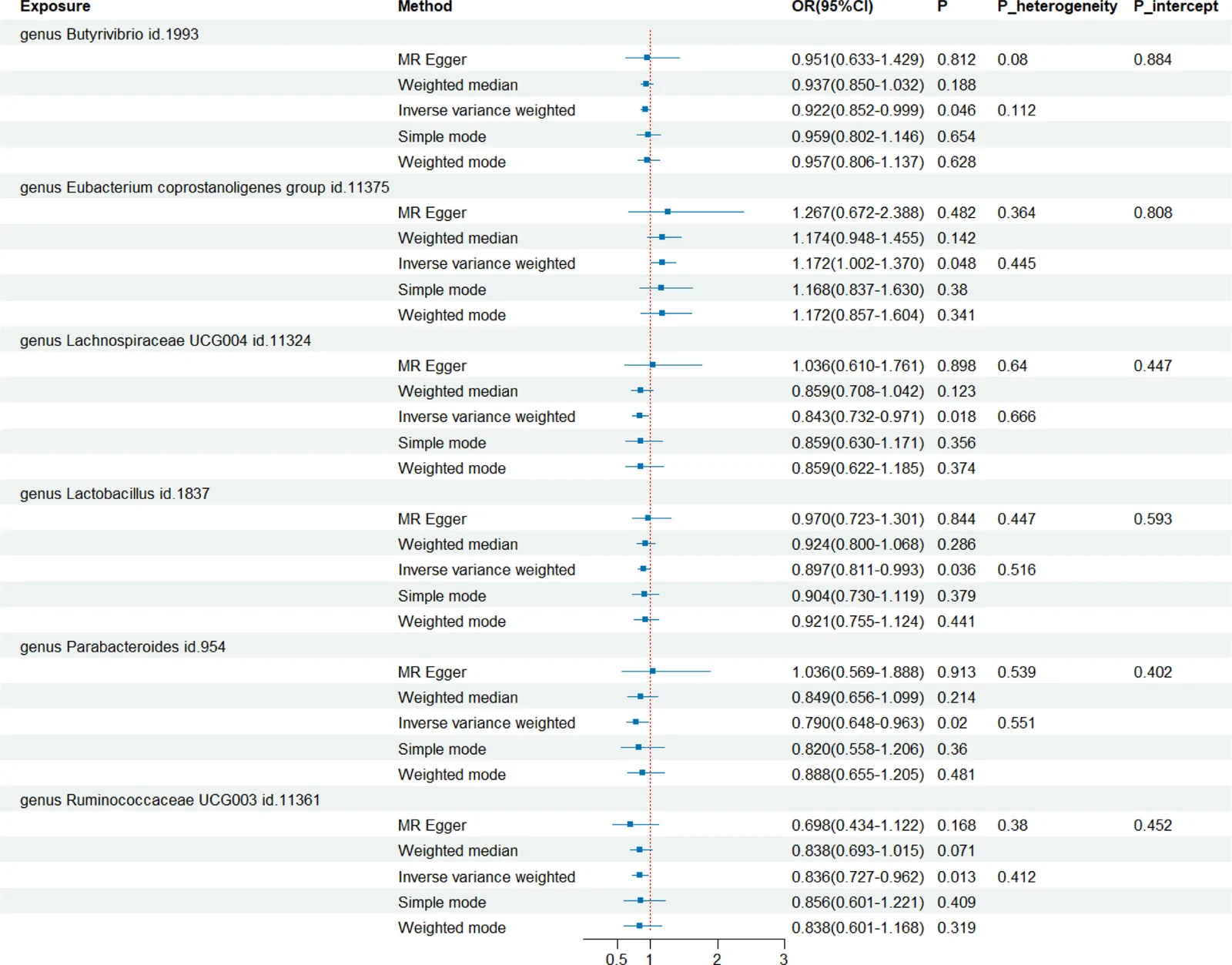
The causal relationship between GM and FS. CI = confidence interval, FS = frozen shoulder, GM = gut microbiota, MR = Mendelian randomization, OR = odds ratio.

**Figure 4.**
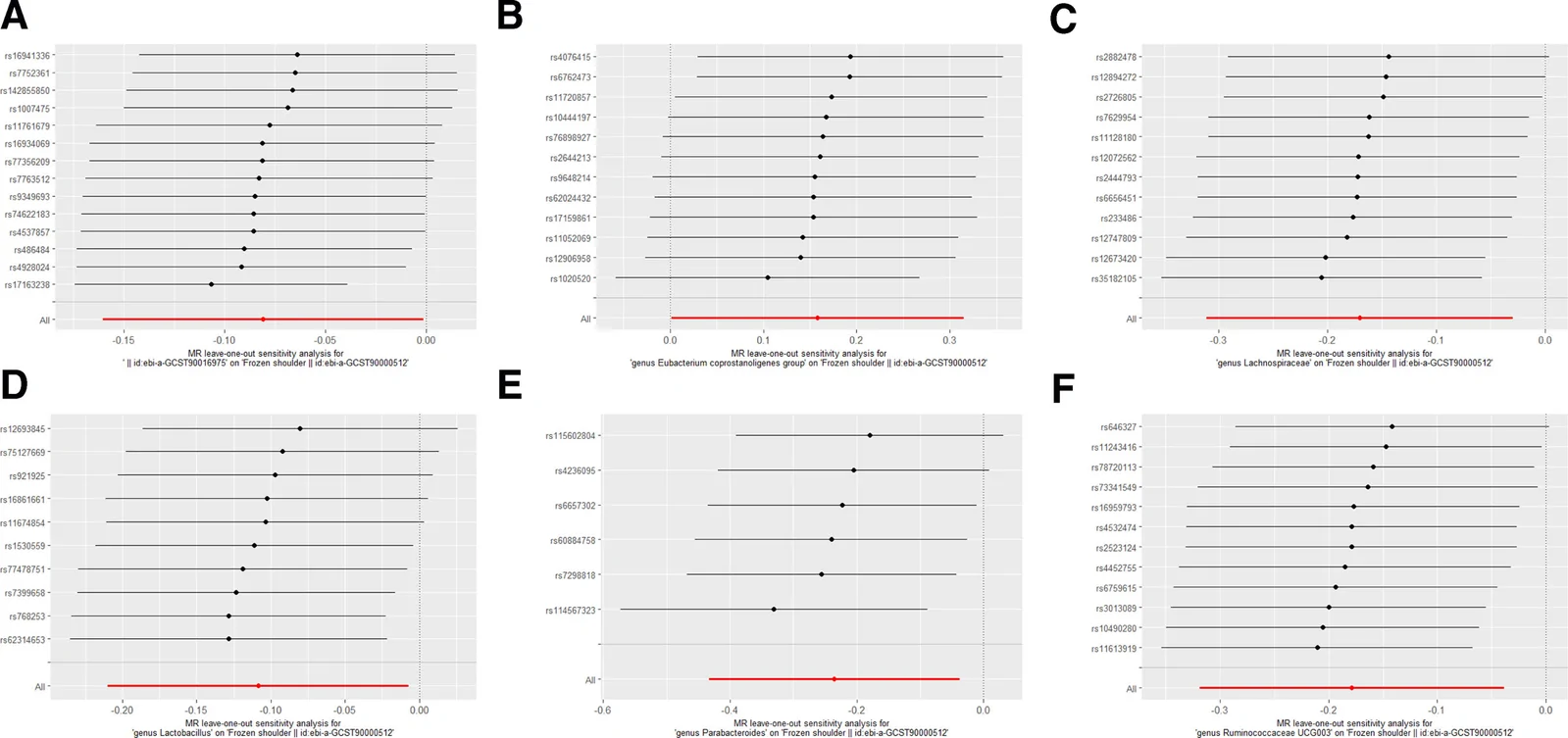
Results of leave-one-out analysis for suggestively causal effects of GM on FS. (A) Forest plots for Butyrivibrio on FS. (B) Forest plots for *Eubacterium coprostanoligenes* on FS. (C) Forest plots for Lachnospiraceae UCG004 on FS. (D) Forest plots for Lactobacillus on FS. (E) Forest plots for Parabacteroides on FS. (F) Forest plots for Ruminococcaceae UCG003 on FS. FS = frozen shoulder, GM = gut microbiota, MR = Mendelian randomization.

Firstly, to ensure the reliability of our results, we retained only 131 genera with a mean abundance >1% in 211 gut microbial taxa, including 131 genera, 35 families, 20 orders, 16 classes, and 9 phyla, and then excluded 12 additional genera that had not been identified. Therefore, only 119 taxonomic units at the genus level were included for analysis in the present study. The 119 genera were used as exposure factors and FS as outcome to perform MR analysis. All IVs were qualified for *F*-statistics > 10 (Supplementary File 3, Supplemental Digital Content, https://links.lww.com/MD/Q159), indicating no evidence of weak instrument bias and our selected IVs were strongly correlated with exposure. Mainly using the IVW approach, we identified 6 suggestive associations between GM and FS, while the effect directions of the weighted median, MR-Egger, weighted mode, and simple mode approaches were consistent, as shown in Figure 3. As shown in the results, in the IVW approach, genus Butyrivibrio (β = −0.081, OR = 0.922, 95% CI = 0.852–0.999, *P* = .046), genus Lachnospiraceae UCG004 (β = −0.171, OR = 0.897, 95% CI = 0.811–0.993, *P* = .036), genus Lactobacillus (β = −0.108, OR = 0.843, 95% CI = 0.732–0.971, *P* = .018), genus Parabacteroides (β = −0.235, OR = 0.79, 95% CI = 0.648–0.963, *P* = .02), genus Ruminococcaceae UCG003 (β = −0.179, OR = 0.836, 95% CI = 0.727–0.962, *P* = .013) were protective factors and inhibited FS. Genus *Eubacterium coprostanoligenes* (β = 0.158, OR = 1.172, 95% CI = 1.002–1.37, *P* = .048) was a risk factor for FS and promoted FS. The same effect trend as IVW was also seen in the results of the weighted median, MR-Egger, weighted mode, and Simple mode method analyses. In the sensitivity analysis, we used Cochran’s *Q*, MR-Egger intercept, MR-PRESSO (Table 1), leave-one-out method (Fig. 4), scatter diagrams (Supplementary File 2, Supplemental Digital Content, https://links.lww.com/MD/Q158) and no heterogeneity or pleiotropy was detected, so our MR results were reliable and accurate.

**Table 1:** Results of heterogeneity and horizontal pleiotropy analysis for IVs of GM associated with FS.

Exposure	Heterogeneity test		MR-Egger intercept test		MR-PRESSO global test	
	IVW	MR-Egger	Egger_intercept	*P*	RSS obs	*P*
Genus Butyrivibrio	*Q* = 19.387	*Q* = 19.3	−0.0043	.884	14.694	.716
	*P* = .112	*P* = .08				
Genus *Eubacterium coprostanoligenes*	*Q* = 10.983	*Q* = 10.915	−0.005	.808	9.381	.519
	*P* = .445	*P* = .364				
Genus Lachnospiraceae UCG004	*Q* = 8.516	*Q* = 7.89	−0.014	.447	9.621	.169
	*P* = .666	*P* = .64				
Genus Lactobacillus	*Q* = 8.177	*Q* = 7.867	−0.009	.593	11.287	.539
	*P* = .516	*P* = .447				
Genus Parabacteroides	*Q* = 3.989	*Q* = 3.11	−0.028	.402	13.618	.716
	*P* = .551	*P* = .539				
Genus Ruminococcaceae UCG003	*Q* = 11.379	*Q* = 10.721	0.139	.452	12.067	.325
	*P* = .412	*P* = .38				

FS = frozen shoulder, GM = gut microbiota, IVs = instrumental variables, IVW = inverse variance weighted, MR = Mendelian randomization, MR-PRESSO = Mendelian randomization-pleiotropy residual sum and outlier, RSS = r esidual sum of squares.

### 3.2. Reverse-direction MR analysis between GM and FS

**Figure 5.**
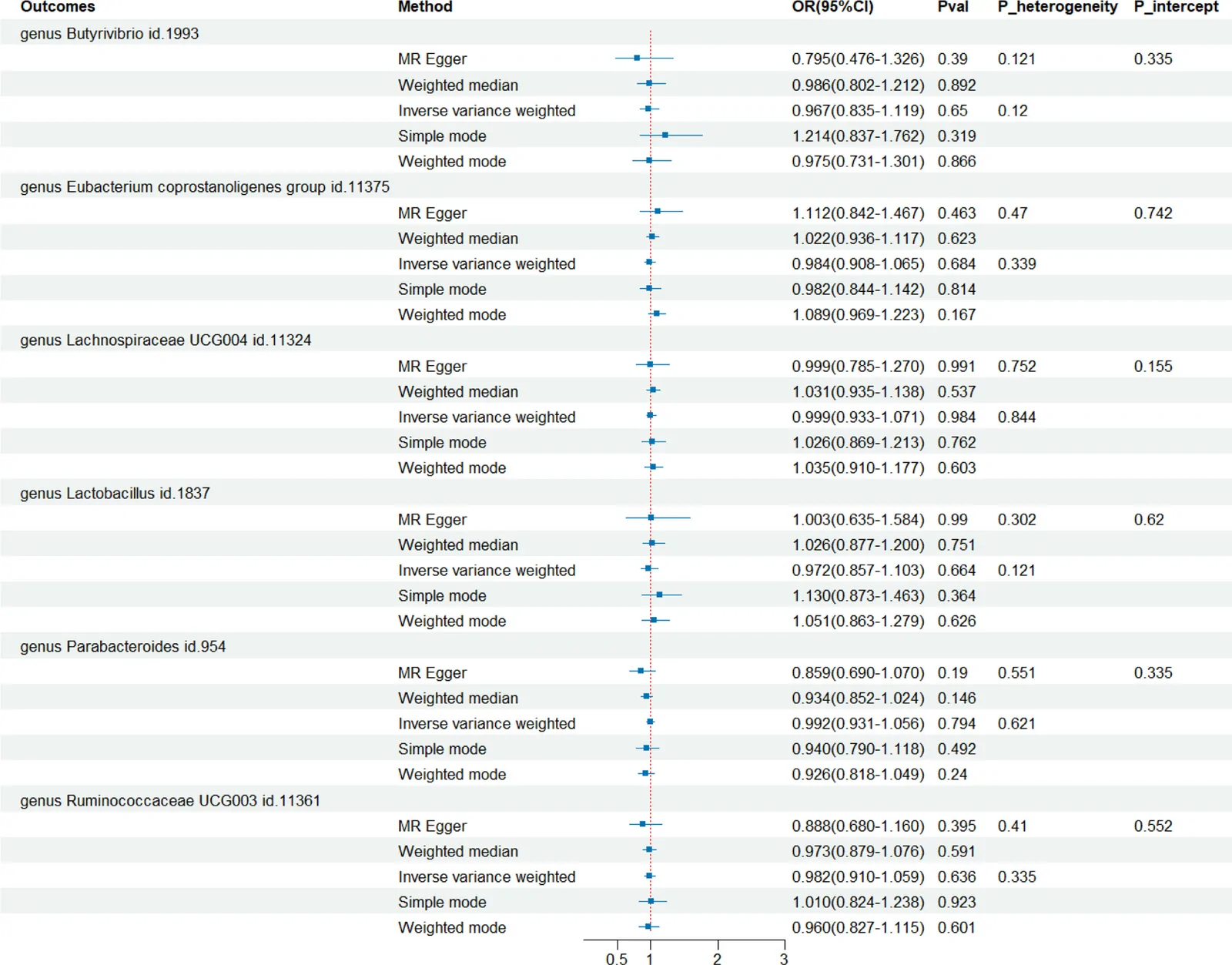
The causal effects between FS and GM. CI = confidence interval, FS = frozen shoulder, GM = gut microbiota, MR = Mendelian randomization, OR = odds ratio.

To verify the directional specificity of the causal association between GM and FS, a further reverse MR analysis was performed to assess the potential reverse causal effect of periostitis on flora composition. Based on the independent IVs screened from the GWAS data of FS, the IVW analysis showed that FS was not significantly causally associated with any of the 6 GM genera mentioned above (Fig. 5). The results were as follows: genus Butyrivibrio (β = −0.034, OR = 0.967, 95% CI = 0.835–1.119, *P* = .65), genus *E coprostanoligenes* (β = −0.017, OR = 0.984, 95% CI = 0.908–1.065, *P* = .684), genus Lachnospiraceae UCG004 (β = −0.001, OR = 0.999, 95% CI = 0.933–1.071, *P* = .984), genus Lactobacillus (β = −0.028, OR = 0.972, 95% CI = 0.857–1.103, *P* = .664), genus Parabacteroides (β = −0.008, OR = 0.992, 95% CI = 0.931–1.056, *P* = .013), genus Ruminococcaceae UCG003 (β = −0.018, OR = 0.982, 95% CI = 0.910–1.059, *P* = .013) showed no horizontal pleiotropy or significant heterogeneity in Cochran’s *Q* and MR-Egger intercept analyses.

### 3.3. Mediating effects of inflammatory cytokines

In the mediation effect analysis of inflammatory factors, this study firstly evaluated the causal effect of GM on 6 candidate inflammatory factors (IL-6, TNF-α, CRP, IFN-γ, IL-1β, IL-10). IVW analysis showed that only genus Lactobacillus had a causal association with IFN-γ (OR = 1.186, 95% CI = 1.039–1.353, *P* = .011) with a causal association (Table 2). Using the IVW estimate, IFN-γ was associated with FS (OR = 0.876, 95% CI = 0.651–0.969, *P* = .021; Supplementary File 4, Supplemental Digital Content, https://links.lww.com/MD/Q158). The results showed that IFN-γ mediated the protective effect of genus Lactobacillus against FS. All other analyses of GM and 6 inflammatory factors were reported in Supplementary File 5, Supplemental Digital Content, https://links.lww.com/MD/Q159. Sensitivity analysis showed no horizontal pleiotropy (MR-Egger intercept *P* > .05) and significant heterogeneity.

**Table 2:** Causal effects between genus Lactobacillus and IFN-γ.

Exposures	Outcomes	SNPs	Method	OR (95% CI)	*P*	*P* heterogeneity	*P* intercept
Genus Lactobacillus	IFN-γ	9	MR-Egger	1.266 (0.778–2.059)	.374	.544	
			Weighted median	1.168 (0.98–1.392)	.092		
			Inverse variance weighted	1.186 (1.039–1.353)	.011	.643	.793
			Simple mode	1.048 (0.763–1.439)	.760		
			Weighted mode	1.045 (0.787–1.388)	.765		

CI = confidence interval, IFN-γ = interferon-γ, OR = odds ratio, MR = Mendelian randomization, SNPs = single nucleotide polymorphisms.

## 4. Discussion

In this study, for the first time, we systematically revealed the causal relationship between gut flora and periostitis by MR and explored the potential mediating roles of 6 inflammatory factors in this relationship. Six bacterial genera (Butyrivibrio, Lachnospiraceae UCG004, Lactobacillus, Parabacteroides, Ruminococcaceae UCG003, and *E coprostanoligenes*) were found to be significantly associated with the risk of FS. Among them, increased abundance of Butyrivibrio (OR = 0.922), Lachnospiraceae UCG004 (OR = 0.897), Lactobacillus (OR = 0.843), Parabacteroides (OR = 0.790), and Ruminococcaceae UCG003 (OR = 0.836) showed a protective effect, whereas the increased abundance of *E coprostanoligenes* (OR = 1.172) was associated with increased risk of FS. The reverse MR analysis further supported the unidirectional causal chain “flora → disease,” and no reverse effect of FS on flora structure was found. Mediation analysis identified IFN-γ as a potential mediator of FS reduction in Lactobacillus.

It is worth noting that most of the protective bacterial genera are SCFAs producers or probiotic-related groups,^[38]^ and their functional characteristics provide important clues to explain their protective mechanisms. For example, the probiotic genera Lactobacillus and Parabacteroides may indirectly reduce systemic inflammatory burden by improving intestinal barrier function and reducing endotoxin translocation.^[39]^ In addition, Butyrivibrio and Ruminococcaceae are major producers of butyrate,^[40]^ which has been shown to promote the differentiation of regulatory Tregs by activating the GPR43 receptor and suppressing Th17 cell-mediated inflammatory responses.^[41]^ In addition, butyrate acts as a histone deacetylase inhibitor, can block the transformation of fibroblasts into myofibroblasts, and reduces collagen deposition.^[42]^ Taken together, these mechanisms suggest that the GM may inhibit the progression of shoulder joint fibrosis through a multidimensional “metabolic-immune-collagen deposition” pathway.

This study found that an increased abundance of *E coprostanoligenes* was associated with an increased risk of shoulder periarthritis, but the specific pathological mechanisms need to be further investigated. This bacterial genus is known to be involved in cholesterol metabolism and the production of secondary bile acids,^[43]^ and bile acids can promote fibroblast activation and collagen synthesis by activating the FXR receptor.^[44]^ This finding suggests that microbial metabolites may promote fibrosis by acting directly on the local microenvironment of the shoulder joint. However, the current study cannot confirm whether bile acids cross the “gut-synovial” barrier and target shoulder joint tissues. Future studies using animal models or in vitro experiments are needed to validate this hypothesis. In recent years, the gut-joint axis theory has emerged as a major research focus in orthopedic diseases, with GM potentially exerting universal regulatory effects in various musculoskeletal disorders.^[45]^ However, studies specifically investigating the impact of GM on the shoulder joint are limited and the underlying mechanisms warrant further investigation.

In recent years, probiotics have emerged as an important intervention to regulate GM and have received increasing attention for their potential in the treatment of musculoskeletal disorders.^[46]^ Although clinical trials of probiotics targeting joints are still in their infancy, preliminary evidence suggests that they may exert therapeutic effects through the “gut-joint axis.” In a collagen-induced arthritis mouse model, oral administration of Pseudocatenulatum suppressed abnormal Th1/Th17-type immune responses by increasing bile salt hydrolase enzyme activity and levels of unconjugated secondary bile acids, thereby restoring intestinal barrier function and altering the composition of the intestinal microbiota to prevent joint damage.^[47]^ In addition, a meta-analysis of probiotic mixtures (containing Bifidobacterium and Lactobacillus) in rheumatoid arthritis showed that they reduced joint swelling and inhibited Th17 cell activity,^[48]^ suggesting that similar mechanisms may be applicable to the treatment of shoulder periarthritis. In this study, MR analysis identified 6 GM taxonomic groups associated with FS. Future studies could further explore animal experiments targeting these 6 bacterial communities as therapeutic targets for arthritis.

In the mediation analysis, the study included classic pro-inflammatory factors in arthritis such as IL-6, TNF-α, and CRP,^[23,49]^ with only IFN-γ showing partial mediating effects. Specifically, increased Lactobacillus abundance may indirectly reduce the risk of shoulder periarthritis by upregulating IFN-γ levels, while factors such as IL-6 and TNF-α did not show significant mediating effects. However, the effects of IFN-γ on fibrosis and collagen deposition remain controversial in the scientific community. A widely accepted view is that IFN-γ has a dual role, depending on disease stage, microenvironment, and interactions with other cytokines. On the one hand, IFN-γ can promote extracellular matrix degradation by upregulating matrix metalloproteinases (MMPs) (such as MMP-1/MMP-13) and inhibiting tissue inhibitor of metalloproteinases, thereby suppressing fibrosis.^[50]^ On the other hand, as a characteristic cytokine of Th1 cells, IFN-γ can promote fibroblast proliferation and collagen synthesis by activating the STAT1 signaling pathway. In an experiment using IFN-γ-pretreated induced pluripotent stem cell-derived mesenchymal stem cells, IFN-γ significantly promoted the secretion of more secretory proteins by hepatocytes, thereby promoting collagen deposition.^[51]^ Notably, IFN-γ has been shown to play a dual role in some fibrotic diseases, such as pulmonary fibrosis. In the early stages, it reduces collagen deposition by inhibiting the TGF-β/Smad3 pathway, while in the late stages, it indirectly exacerbates fibrosis by upregulating the expression of factors such as TNF-α.^[52]^ However, whether IFN-γ plays an important positive role in the process of Lactobacillus alleviating arthritis requires further research and verification. Nevertheless, this finding provides new insights into the field of probiotic therapy for arthritis-related conditions. It is currently unclear how probiotics inhibit or cure arthritis; however, other researchers have previously observed reduced intestinal permeability, which is associated with immune system regulation. This regulatory activity may be related to improved local secretion of IgA inflammatory cells toward pathogens, reduced pathogen proliferation, and downregulation of immune response components such as IL-12 and TNF-α.^[53]^ Animal models of RA indicate that oral probiotics can reduce intestinal permeability, thereby alleviating the severity of arthritis.^[54]^ A recent clinical trial evaluated the efficacy of oral probiotic *Lactobacillus rhamnosus* treatment. Compared with the placebo group, RA patients who took oral probiotic *L rhamnosus* GR-1 and *Lactobacillus reuteri* RC-14 capsules showed improved joint function.^[55]^

This study has several limitations that should be carefully considered. Firstly, although MR effectively avoided confounding, genetic IVs can only explain part of the variation in microbiota (with an average heritability of about 5–15%) and may miss nongenetic regulation of microbiota.^[56]^ Secondly, after Bonferroni correction, the *P*-values of the 6 selected microbial communities ranged from.05 to 4.2 × 10⁻^4^, suggesting a potential association between the 2, which should be interpreted with caution. However, the biological conclusions of this study are consistent with those of numerous other studies in the field of the gut-bone axis,^[39,42,45,55–57]^ thereby increasing the credibility of the study’s findings. Thirdly, GWAS data is mainly from European populations, and conclusions should be verified when extrapolating to other populations. In addition, previous studies have shown that the onset of FS is associated with age and sex,^[1]^ with a higher incidence in women around the age of 50. However, more specific individual data were not available, and we were unable to perform more detailed subgroup analyses by age or sex. In addition, the mediation analysis included only a subset of serum inflammatory markers and did not include joint fluid- or tissue-specific markers, which may underestimate the role of inflammatory pathways and miss inflammatory markers with potential mediating effects. Future studies could utilize multi-omics integration, such as metagenomics, metabolomics, and single-cell transcriptomics, to elucidate the spatial specificity of microbiota-host interactions. Furthermore, fecal microbiota transplantation targeting significant bacterial genera or supplementation with specific probiotics could help validate their causal effects and clinical translation potential.

## 5. Conclusion

This study demonstrated a causal association between GM and shoulder periarthritis through MR. These findings not only expand the theoretical scope of the “gut-joint axis” but also provide new targets for targeted prevention and treatment of shoulder periarthritis. Future studies should combine experimental models with clinical cohorts to further elucidate the molecular mechanisms underlying microbiota regulation of shoulder joint pathology and explore personalized intervention strategies targeting microbiota.

## Acknowledgments

The authors thank all participants for the study that they conducted.

## Author contributions

**Conceptualization:** Zhiyun Feng.

**Data curation:** Zhiyun Feng.

**Formal analysis:** Zhiyun Feng.

**Funding acquisition:** Zhiyun Feng.

**Investigation:** Zhiyun Feng.

**Resources:** Zhiyun Feng.

**Software:** Zhiyun Feng.

**Supervision:** Tao Ding.

**Visualization:** Xiule Fang.

**Writing – original draft:** Zhiyun Feng, Xiule Fang.

**Writing – review & editing:** Zhiyun Feng.

## Supplementary Material
