# Supplementary material for: Effect of the gut microbiota on frozen shoulder and potential mediating role of inflammatory cytokines: A Mendelian randomization study and mediation analysis
Source: Medicine (Baltimore). 2025 Sep 26;104(39):e44595. doi: 10.1097/MD.0000000000044595 (PMC13593230; doi:10.1097/MD.0000000000044595)
Supplement: Supplementary file 1 [file medi-104-e44595-s001.pdf]

Supplementary file.1 GWAS data information for inflammatory cytokines

Variable	GWAS ID	Sample size	Number of SNPs
IL-6	ebi-a-GCST004446	8189	9790590
IL-8	ebi-a-GCST004445	3526	9517348
IL-10	ebi-a-GCST004444	7681	9793415
TNF-α	ebi-a-GCST90012044	21,758	12,713,951
CRP	ebi-a-GCST90029070	575,531	10,713,245
INF-γ	ebi-a-GCST004456	7701	9785363

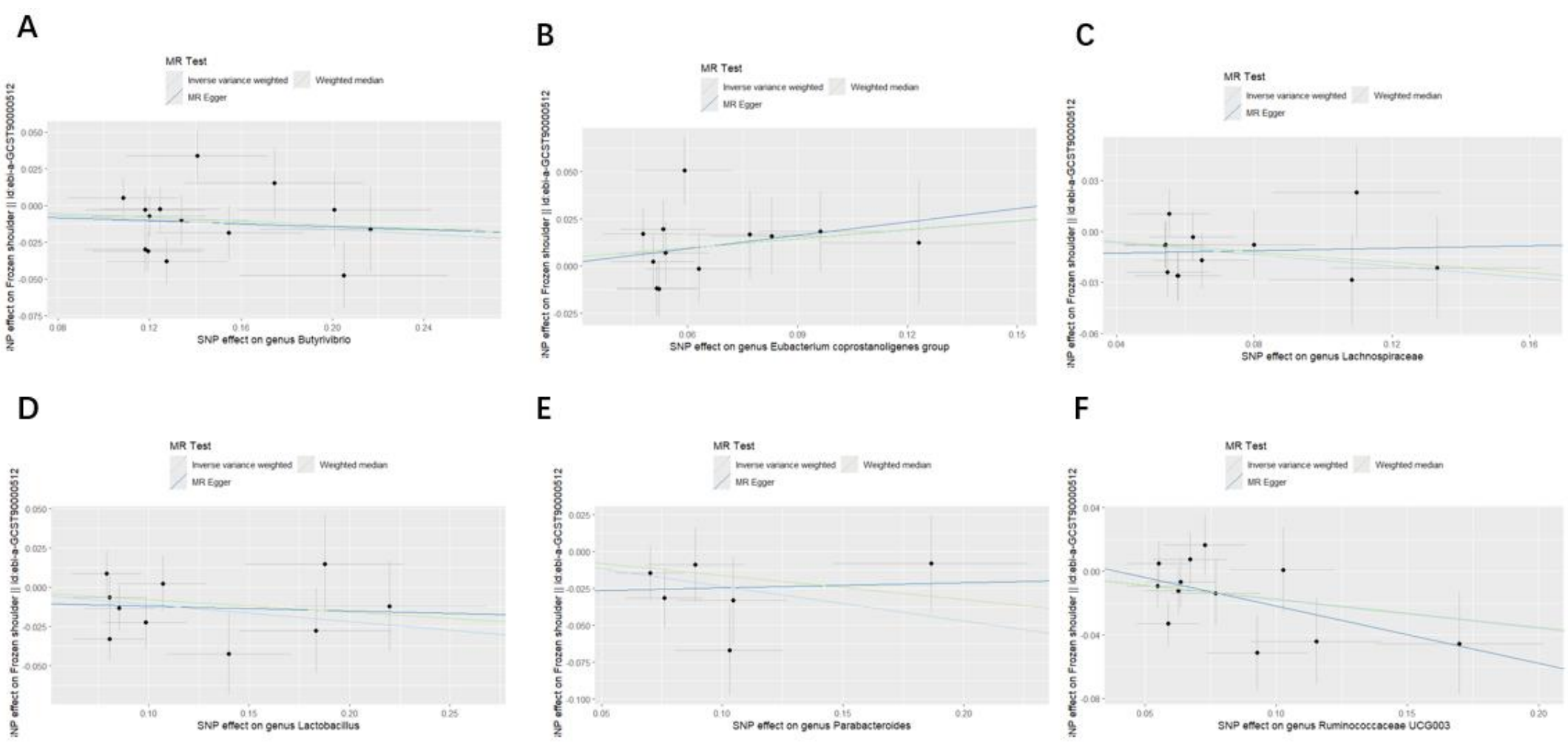

Supplementary file.2 The scatter plots for causal effect of GM on FS. **A**) Butyrivibrio on FS. **B**) Eubacterium coprostanoligenes on FS. **C**) Lachnospiraceae UCG004 on FS. **D**) Lactobacillus on FS. **E**) Parabacteroides on FS. **F**) Ruminococcaceae UCG003 on FS.

Supplementary flie 4 Causal effects between IFN-γ and Frozen Shoulder.

Exposures	Outcomes	SNPs	Method	OR (95% CI)	P	P heterogeneity	P intercept
IFN-γ	FS	12	MR Egger	0.974（0.741-1.163）	0.716	0.637	
			Weighted median	0.951（0.819-1.027）	0.216		
			Inverse variance weighted	0.876（0.651-0.969）	0.021	0.732	0.734
			Simple mode	0.875（0.633-1.115）	0.063		
			Weighted mode	0.986（0.816-1.057）	0.135		
